# On the Use of the Neglected Edible Plants *Soda inermis* Fourr. and *Bunias erucago* L. on the Adriatic Coast of Croatia

**DOI:** 10.3390/plants15142206

**Published:** 2026-07-19

**Authors:** Ivana Vitasović-Kosić, Marija Jug-Dujaković, Katija Dolina, Tonka Ninčević Runjić, Łukasz Łuczaj

**Affiliations:** 1 Department of Agricultural Botany, Division of Horticulture and Landscape Architecture, Faculty of Agriculture, University of Zagreb, Svetošimunska Cesta 25, 10000 Zagreb, Croatia; ivitasovic@agr.hr; 2 Institute for Adriatic Crops and Karst Reclamation, Put Duilova 11, 21000 Split, Croatia; masagatin@gmail.com (M.J.-D.); tonka.nincevic.runjic@krs.hr (T.N.R.); 3 Institute for Marine and Coastal Research, University of Dubrovnik, Kneza Damjana Jude 12, 20000 Dubrovnik, Croatia; katija.dolina@unidu.hr; 4 Faculty of Biology, Nature Protection and Sustainable Development, University of Rzeszów, Pigonia 1, 35-310 Rzeszów, Poland

**Keywords:** ethnobotany, wild crop relatives, vegetables, brassicas, Mediterranean diet

## Abstract

Background: Between 2012 and 2023, extensive fieldwork was carried out in coastal Croatia to document traditional plant use. Among the wild vegetables collected, we noted two edible species of leafy greens that are both wild-gathered and cultivated. These are *Soda inermis* Fourr. (syn. *Salsola soda* L., Amaranthaceae) and *Bunias erucago* (Brassicaceae). Methods: The aim of this paper was to review their use in Croatia, both based on the published data and our unpublished observations, altogether encompassing 502 interviews on wild food use. Results: *S. inermis* was recorded in 11 localities (20 interviews). Although mainly cultivated, it is gathered from the wild and sold at vegetable markets on a few islands of northern Dalmatia and near Zadar. *B. erucago* was recorded in 40 localities (78 interviews). Used chiefly in central and southern Dalmatia as well as on a few adjacent islands, it is usually collected from the wild, more rarely cultivated. Conclusions: *S. inermis* represents a declining tradition of cultivation that is closely tied to specific ecological niches and worth preserving. The more resilient *B. erucago* is either a neglected crop or an example of incipient domestication. Cultivation trials should be undertaken to establish its potential as a future crop.

## 1. Introduction

Over the last twelve thousand years, apart from the use of wild vegetables, humanity has developed a diversity of cultivated crops. The number of cultivated species and varieties has been in decline for a few decades. A certain number of species has been only incipiently cultivated or supported by the maintenance of semi-natural human controlled landscapes such as open grazed woodland or fire-dependent ecosystems [1,2,3]. Due to climate change, it is important to monitor wild crop relatives and their genetic resources [4,5].

Neglected crops are defined as species that have received little attention from research and development, yet continue to be used locally, where they remain well adapted and competitive [6]. Many such species, although minor at a global scale, play important roles in regional food systems, particularly in marginal environments. Despite their potential contribution to food security and agrobiodiversity, they are often poorly documented, with limited accessible information and weak conservation efforts. This lack of coordinated research and awareness has hindered their wider utilization and sustainable management [6].

Between 2012 and 2023, extensive fieldwork documenting traditional uses of wild food plants was carried out in coastal Croatia. A part of the results has already been published, but some interesting data are still being analyzed [7,8,9,10]. Among the species we studied, we observed two, namely (*Bunias erucago* L.) and *Soda inermis* Fourr. (syn. *Salsola soda* L.), which are both collected from the wild and cultivated there as well as in other parts of the Mediterranean (for plant names, we followed Plants of the World Online). As these species form a disappearing or little-known part of local culinary heritage, they merit special attention.

*B. erucago* L. is a Mediterranean Brassicaceae species, widely distributed across southern Europe [11]. Traditionally used as a leafy vegetable, it has been documented in several ethnobotanical studies across southern Europe, the Balkans and even the Caucasus, e.g., in Italy [12,13,14,15], Croatia [16], Bosnia and Herzegovina [17] and Georgia [18,19,20]. Although typically collected from the wild, cases of its small-scale cultivation have been reported in Italy, indicating its status as a neglected or underutilized crop [13,21]. The species is of particular interest due to its pleasant taste and high nutritional value, including significant levels of β-carotene, vitamin E and polyphenols, as well as its potential role as a nutraceutical plant [21,22].

Across the Italian Peninsula, Dalmatia and Herzegovina, the basal leaves of these taxa are traditionally harvested from the wild prior to flowering and consumed either boiled and seasoned with oil or butter or eaten raw in salads [7,17,23,24,25]. Their use is sometimes linked to specific local recipes and occasional small-scale cultivation. However, the literature provides inconsistent accounts regarding their present role in culinary traditions and their cultivation status [26,27].

*B. erucago* has been cultivated on a small scale in the Lomellina area (Po Plain, northern Italy) and used to make a rice soup called “ris e barlánd” [13,26,28,29]. Propagation relies largely on locally sourced or wild-collected seeds, sometimes maintained through self-sowing. However, over recent decades, the species has become increasingly rare in the wild, likely due to agricultural intensification and herbicide use [13,24,26,30]. In Italy, wider cultivation is limited by poor and irregular germination [21].

*S. inermis* Fourr., formerly known as *Salsola soda* L. (Amaranthaceae) is an annual halophytic species widely distributed across coastal and saline habitats of the Mediterranean Basin, including Italy and the eastern Adriatic coast of Croatia, as well as some inland localities, e.g., the salt marshes of Hungary [31]. The species is highly adapted to extreme environmental conditions such as high salinity, intense solar radiation, and drought and typically occurs on sandy and gravelly coastal substrates and salt-affected soils [32]. As a light-demanding halophyte, it is characteristic of Mediterranean coastal ecosystems and contributes to the stability and functioning of saline habitats. More broadly, species of the genus *Salsola* are typical of arid and semi-arid regions and exhibit strong tolerance to abiotic stress factors [33].

Historically, *S. inermis* played a major economic role in Mediterranean Europe, particularly in Italy and Spain, where it was cultivated for the production of “barilla,” a sodium carbonate-rich ash used in glassmaking and soap production, but was also eaten as a vegetable, especially in Italy [34,35,36,37,38]. Its cultivation on saline soils unsuitable for conventional crops highlights its long-standing importance in marginal agricultural systems [39]). This use declined with the introduction of industrial soda production.

In contemporary Italy, *S. inermis* remains a well-known, traditionally used, edible halophyte, commonly referred to as “agretti,” “barba di frate,” or “roscano” [6,23,35]. Ethnobotanical studies from southern Italy, particularly Apulia, document its continued use as a seasonal wild or semi-cultivated vegetable [39]. Young shoots are typically harvested in spring and prepared by boiling or steaming, then consumed with olive oil, lemon juice, or incorporated into pasta dishes and salads [39,40]. In some areas, the plant is also sautéed or used as a filling ingredient, reflecting diverse culinary traditions rooted in local ecological knowledge [40]. Similarly, studies from Tuscany identify *S. inermis* among ethnobotanically important halophytes in coastal brackish systems, where it is both collected from the wild and increasingly considered for local cultivation [41]. In Greece, where it is called *almyra*, the species is widely known in coastal areas. It is even served in taverns. It is usually boiled and seasoned with lemon like other *horta* species, although it can be used in a variety of other Greek dishes [42,43]. Surprisingly, the broad ethnobotanical literature on the use of wild food plants in Turkey does not mention it [44].

In Croatia, *S. inermis* was recorded as sold in the vegetable markets of Zagreb and Šibenik [32] as well as in Zadar [45]. The use of *S. inermis* was also reported on Pag by Łuczaj et al. [12] and on Zlarin by Viculin et al. [46].

Nutritionally, *S. inermis* is valued as a low-calorie vegetable reported to be rich in vitamins and minerals, including vitamins A, C, and K [32]. This nutritional profile, combined with its unique taste and cultural significance, supports its role as a functional food within Mediterranean diets. In Croatia, similar uses have been documented, where the species is recognized as a wild edible plant and is increasingly utilized and cultivated along the Adriatic coast [32].

Beyond food use, species of the genus *Salsola* are known for their ethnomedicinal relevance. They contain bioactive compounds such as alkaloids and phenolics with antioxidant, antimicrobial, anti-inflammatory, and antihypertensive properties [47]. Although *S. inermis* is less prominent medicinally than other congeners, it has been reported to possess mild diuretic and digestive properties in traditional use [35].

Ecologically, *S. inermis* contributes to soil stabilization, plays a role in the restoration of saline and degraded lands [12,33,47,48], and can improve the performance of companion plants [49].

In Mediterranean socio-ecological systems, particularly in Italy and Croatia, *S. inermis* represents a multifunctional species that connects ecological adaptation, traditional knowledge, and economic opportunity. Its continued presence in local diets, emerging role as a sustainable crop, and ecological importance in coastal habitats underscore the species’ relevance in contemporary ethnobotanical research. The conservation and sustainable management of this species are therefore essential for maintaining both biodiversity and Mediterranean biocultural heritage in the face of environmental and socio-economic change.

*S. inermis* has the status of a vulnerable plant species (VU) according to the Croatian Red List in Croatia. It is also listed as a vulnerable species in Slovenia and as a rare species in Hungary [50] due to the reduction in the area and/or quality of its habitat, human settlements, tourism and infrastructure (roads, embankments, power lines) [32].

The aims of this study were: (1) to document the current distribution and culinary use of *Bunias erucago* and *Soda inermis* in coastal Croatia; (2) to assess their status along the wild–cultivated continuum; and (3) to discuss their relevance as neglected or underutilized crops. Overall, the paper is an ethnobotanical synthesis combining published records, unpublished field observations, interview data, and market observations.

## 2. Results

### 2.1. Distribution and Use of Bunias erucago

**Figure 1:**
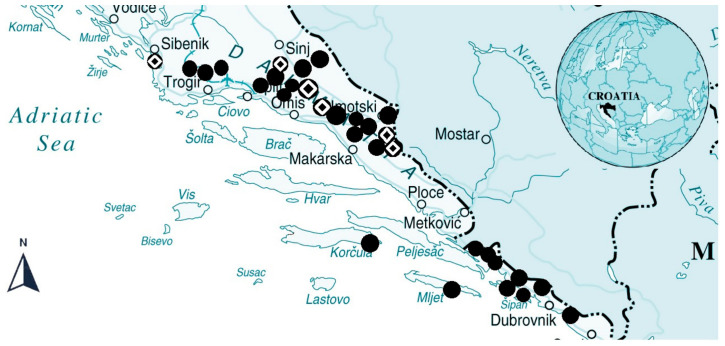
Map of *Bunias erucago* use in Croatia. The black dots mark places where the species is only collected from the wild; the white rhomboids—areas where the species is cultivated (the base map was adapted from the map of Croatia prepared by the UN Department of Peacekeeping Operations, Cartographic Section, and is used under the Creative Commons Attribution-ShareAlike 3.0 (CC BY-SA 3.0) licence) [51].

We recorded the use of *B. erucago* in 78 interviews from 40 settlements throughout Dalmatia. *B. erucago* is widely collected from the wild (Figure 1; Table 1), chiefly in mainland Dalmatia around Split and Dubrovnik, in the Dalmatian Zagora, the Poljica region and on the Dubrovnik coast. It is also used on five islands: Korčula, Šipan, Mljet, Lopud and Krapanj. In the Dubrovnik area, Korčula, Šipan, Mljet and Lopud *B. erucago* is called *pakolić* or *pakoleć*, while in inland Dalmatia, including Poljica and on Krapanj, it is referred to as *grzdulja*. A voucher specimen of *B. erucago* was deposited in the herbarium of Warsaw University (WA000037037).

**Figure 2:**
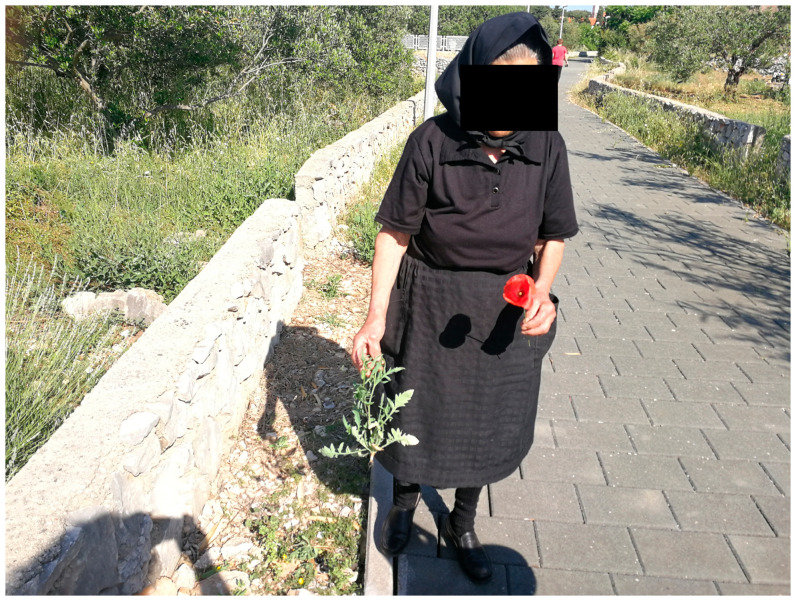
An 84-year-old farmer on the island of Krapanj, who cultivates *Bunias erucago* in the field on the left, holding the species in her right hand (photo by Łukasz Łuczaj, 2018).

**Figure 3:**
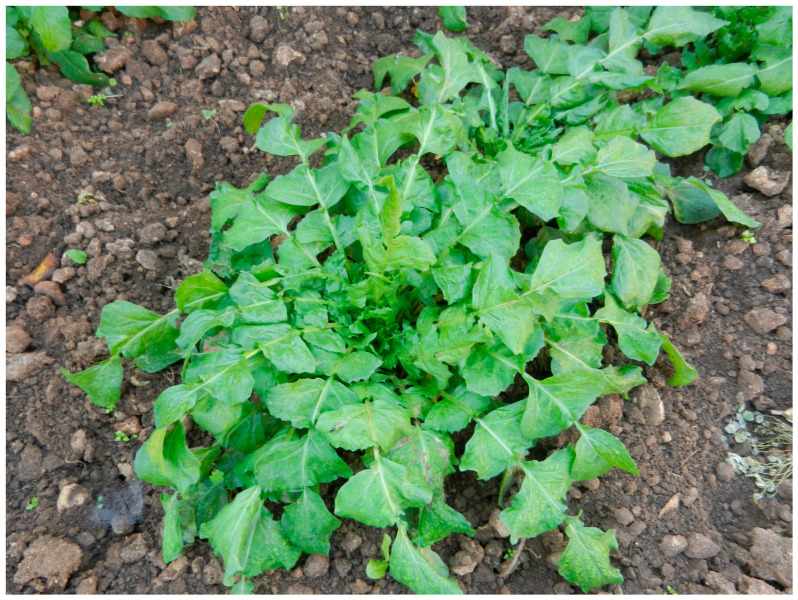
*Bunias erucago* cultivated in the village of Dicmo (photo by Marija Jug-Dujaković, 2016).

We found six informants confirming the cultivation of *B. erucago* (on Krapanj and in Split–Dalmatia County in Seoca, Srijane, Dicmo, Zmijavci and Runovići—see Figure 2 and Figure 3). The species requires little management—sometimes it is simply spared from cutting or its seeds are scattered on the edges of ploughed gardens.

*B. erucago* is usually briefly cooked, then strained and served with olive oil and salt. Sometimes roots are eaten together with the leaves. *B. erucago* may also be cooked with dried meat or broth. In the Dalmatian Zagora, it is sometimes served as a side-dish for polenta (*pura*).

*B. erucago* shows a wide and more continuous distribution of use, especially in central and southern Dalmatia (Figure 1). The dataset includes numerous records from mainland localities within inland Dalmatia (e.g., Dicmo, Klis, Imotski region) and extends southward to the Dubrovnik area, as well as to several islands (Korčula, Mljet, Šipan, Lopud and Krapanj). *B. erucago* was recorded in at least three interviews in the following localities: Cista Provo, Dicmo (Kraj, Osoje), Imotski and Vinjani Gornji, Klis, Lovreć, Ljubitovica (Munizi) and Podbablje, as well as in Šipan and Krapanj.

### 2.2. Distribution and Use of Soda inermis

**Figure 4:**
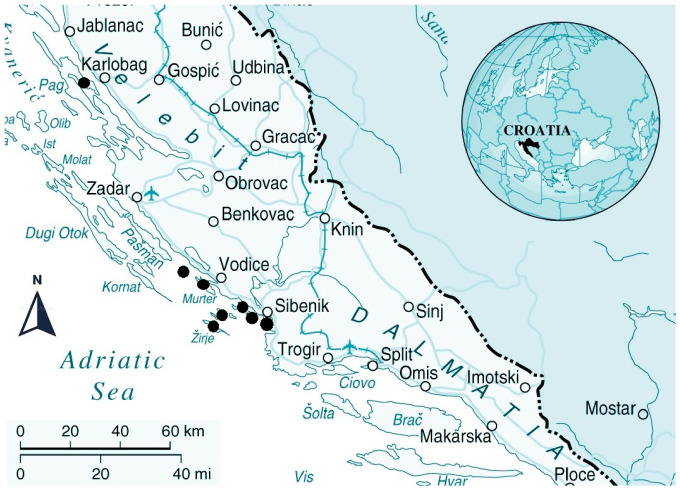
Map of *Soda inermis* traditional use in Croatia. Black dots represent localities where the species is used The base map was adapted from the map of Croatia prepared by the UN Department of Peacekeeping Operations, Cartographic Section, and is used under the Creative Commons Attribution-ShareAlike 3.0 (CC BY-SA 3.0) licence) [51].

The use of *S. inermis* was documented primarily on smaller islands in northern–central Dalmatia near Šibenik (Vrgada, Prvić, Karprije, Žirje, Krapanj), in Zadar (Ražanac) and on the island of Pag (Figure 4). *S. inermis* is called *smucanj* (Vrgada, Murter), *osmukalj* (Kaprije, Prvić, Zlarin), *osmuhalj* (Zlarin), *roška* (Zadar area) or *rosica* (Pag). All the localities where it is used are listed in Table 2. A voucher specimen of *S. inermis* was deposited in the herbarium of Warsaw University (WA0000066392). Altogether, the use of the plant was reported in 20 of our interviews from 10 settlements, as well as in a publication from the island of Zlarin [46].

**Figure 5:**
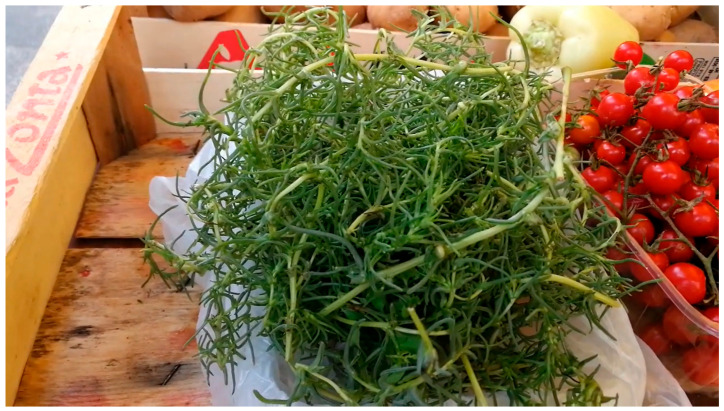
*Soda inermis* sold in Zadar market by a farmer from Ražanac (photo by Łukasz Łuczaj, 2018).

The plant is usually briefly boiled, though it can be eaten raw as a salad with olive oil. It is also eaten with beans. Field observations show that the species is both collected from the wild and cultivated in small household plots that are typically immediately adjacent to the seashore. The plant is sown in late autumn and can be harvested from late spring to early autumn. This ecological proximity appears essential, as the species thrives in saline coastal habitats. Cultivation was confirmed by five respondents, as small-scale and usually for household consumption, in the islands of Murter, Prvić and Žirje, and in Ražanac near Zadar (Table 2). The excess was sold in Šibenik [32] and Zadar markets (Figure 5). *S. inermis* also appears on sale in the capital of Croatia, Zagreb [32].

## 3. Discussion

### 3.1. Comparison of the Use of B. erucago and S. inermis

The two species differ in several key aspects (Table 3). *S. inermis* is strongly associated with coastal microhabitats, occasionally cultivated, and locally marketed in northern Dalmatia. *B. erucago* is widely distributed across central and southern Dalmatia, primarily collected from the wild, and only rarely cultivated. These differences suggest distinct ecological, cultural, and possibly historical drivers of use.

The geographical range of the two species’ use overlaps, but the uses themselves largely do not. Only on Krapanj are both species used. The relatively low number of people using *S. inermis* in each locality suggests that the plant is not intensively consumed even within its range, and knowledge about its use may be fragmented and weakly transmitted.

In contrast, *B. erucago* was mentioned by three and a half times (78) as many respondents as *S. inermis* (20). The species is associated with a much larger number of localities, in many of which it was recorded in multiple interviews. While *S. inermis* is limited to coastal saline habitats, *B. erucago* can be found in inland Dalmatia. Its larger abundance makes it easier to preserve the tradition of its use.

### 3.2. Why Is the Use of Soda inermis Restricted to Smaller Islands?

The use of *S. inermis*, restricted mainly to smaller islands, is an interesting issue; this pattern may be associated with the proximity of smaller settlements to the sea and inhabitants’ permanent contact with it. The species grows in the wild or is cultivated only directly by the sea. Coastal settlements on larger islands are more impacted by tourism. *S. inermis* can be found throughout the coastline of Croatia, but the fact that the species is used only in northern and central Dalmatia, although it can also be found further north in the Kvarner and further south [31], shows that the species’ area of use may be culturally restricted.

### 3.3. Why Is Bunias erucago Used Only in Central and Southern Dalmatia?

*B. erucago* is used only in central and southern Dalmatia. The furthest western locality of its use is the island of Krapanj. The restriction to this part of the coast can possibly be explained by the species’ distribution—*B. erucago* is very abundant east of Split all the way to Dubrovnik [11]) but relatively rare further north. Still, a question arises: maybe the species is abundant there because it was cultivated in the past? Unfortunately, we do not have enough data to establish whether this is true. These two drivers may be working synergistically. All in all, the relatively high number of use-reports for *B. erucago* (*n* = 78), combined with repeated citations from the same localities, indicates that the species is deeply embedded in local food traditions. This strong cultural persistence contrasts with its limited representation in formal agriculture. The documentation of active cultivation on Krapanj and in inland Dalmatia (e.g., Poljica region) is particularly significant. Although rare, this practice demonstrates that the species is amenable to cultivation and may represent a continuum between wild gathering and domestication. Similarly, to the Po Plain in Italy, *B. erucago* can be treated as a neglected crop plant species, given its continued local use, limited scientific attention, and marginal but persistent cultivation [6,13]. One of the key constraints to its wider cultivation is its poor germination performance, which has been experimentally demonstrated. Seeds enclosed in indehiscent silicles show extremely low germination rates (<10%), while mechanical treatments such as scarification or seed extraction significantly improve germination [21]. This physiological constraint explains why the species remains largely wild-collected despite its culinary value.

It must be emphasized that *B. erucago* is an important component of wild vegetable mixes in central Dalmatia. According to Krželj and Vitasović-Kosić [52], a boiled mix of *B. erucago* and other wild vegetables and cabbage leaves is traditionally served on Good Friday in the villages of Šestanovac and Žeževica (Split–Dalmatia County).

Traditional practices observed in our fieldwork, such as allowing plants to self-seed or maintaining small, cultivated patches, are consistent with strategies described elsewhere to overcome germination limitations [21]. These practices represent a form of low-intensity management, typical of incipient domestication processes. A continuum between using a species from the wild and its cultivation has been observed for many Brassicaceae species [53,54].

The documented cases of cultivation in Croatia, although rare, are particularly significant when interpreted in light of agronomic studies. Experimental evidence suggests that relatively simple treatments, such as scarification of the dispersal units, can substantially increase germination success and make cultivation more feasible [21]. This indicates that the current marginal status of *B. erucago* is not due to a lack of agronomic potential, but rather to technical barriers that have not yet been widely addressed in traditional or modern agriculture. The variability in germination performance among populations further suggests that selection of suitable genotypes could play an important role in future domestication efforts [21].

Therefore, *B. erucago* represents a promising candidate for the development of new or revived crops in Mediterranean regions, particularly in the context of climate change and the need for resilient, low-input food resources. In the central Po Plain, particularly within the province of Pavia, *B. erucago* appears to have maintained a stable role in local culinary traditions [13]. Recent indicators—including its celebration in local festivals, its mention in contemporary cookbooks and media, and the limited commercial availability of its seeds from regional seed suppliers—suggest a renewed, albeit small-scale, interest in this species [13]. This pattern aligns with broader trends observed in several countries, where neglected and traditional crops are being rediscovered in connection with local food heritage and marginal agricultural systems [55]. Such persistence is noteworthy given the severe genetic erosion reported for northern Italy, where over 90% of landraces and minor crops have disappeared in recent decades [34].

In contrast to several other native leafy green vegetables formerly cultivated in Italy, now largely restricted to foraging from the wild and no longer considered crops, e.g., *Blitum bonus-henricus*, *Glebionis coronaria*, *Reichardia picroides*, *Rumex scutatus* and *Smyrnium olusatrum* [23], *B. erucago* continues to be actively cultivated there [13]. Time will tell what happens in Dalmatia—whether *B. erucago* will only be collected from the wild or also cultivated. The trajectory of future change will depend on the persistence of wild populations (nowadays, many ruderal weed populations collapse) and whether interest in the cultivation of this crop will be reignited.

### 3.4. Limitations

We treat our study as a preliminary report. The geographic sampling was uneven as it did not cover some parts of the mainland coast. The data were gathered during a study on wild foods, so some interviewees may not have mentioned cultivated plants. Additionally, not all of the use-report should be interpreted as direct measures of current consumption frequency.

## 4. Conclusions

Both species illustrate the importance of wild and semi-domesticated vegetables as components of agrobiodiversity. *S. inermis* represents a declining tradition of cultivation that is closely tied to specific ecological niches. *B. erucago* is a more resilient but regionally bounded resource with potential for further expansion. The species may be a forgotten crop or an example of incipient domestication. In the context of climate change and the search for resilient crops, both species are of interest as wild crop relatives and underutilized vegetables. Their tolerance to marginal conditions (e.g., salinity in *S. inermis*), established culinary use and, in the case of *B. erucago*, disturbed ground make them promising candidates for diversification of local food systems. There is also an urgent need for ethnobotanical documentation of the use of *S. inermis* in Greece, where the species is well-known as a vegetable.

Further research should focus on agronomic experiments, particularly for *B. erucago* (concerning its seed viability), nutritional and phytochemical analyses, and detailed historical studies to clarify the extent of past cultivation. Preserving and revitalizing knowledge associated with these species is essential not only to biological diversity but also to cultural heritage.

## 5. Materials and Methods

This study forms part of larger research on the ethnobotany of the Adriatic islands and Dalmatia, from which data about the use of the two species have been extracted. The research was performed between 2012 and 2023, with most interviews carried out between 2015 and 2017 and between 2022 and 2023. The general data from larger islands and the mainland areas between Split and Dubrovnik were published. The researched species were not used in some studied pericoastal regions, such as the Biograd area [8], Knin area [56] or in Istria [57]. From our work in the regions where *B. erucago* and *S. inermis* have been used, we made a database of 502 interviews from published data: 40 in the Dubrovnik area [16], 67 in the “Poljica Republic” [9]; 170 in the Dalmatian Zagora [58], 225 from 15 largest Adriatic islands [59] and 115 unpublished interviews from 21 smaller Adriatic islands. Data from Šestanovac and Žeževica (13 interviews in which *B. erucago* was mentioned were recorded and published in Krželj and Vitasović-Kosić [52]). In this database, we gathered 78 instances of *B. erucago* use and 23 of the use of *S. inermis*. This also includes our observations from Dalmatian vegetable markets (1 interview in Zadar) and other published data [32,46].

We applied the standard methods of ethnobotany: in-depth semi-structured interviews starting from freelisting, and supplemented, if possible, by walks around the places where the respondents would gather plants to identify the supplied names [60,61,62]. The interviews were performed in Croatian, the native language of the inhabitants. The interviews concerned different aspects of plant use, but here we present data only on *B. erucago* and *S. inermis*. The interviewed people were found either via snowball method or through opportunistic sampling (haphazard encounters with older, middle-aged or elderly inhabitants working in fields and orchards). Oral consent was obtained. A use report was defined as one mention of a species use by one informant or group of informants interviewed together (although mostly individuals were interviewed). The same field protocol was used throughout the study. In most cases, plants were identified using specimens shown to us by the informant.

Plant names were given according to Plants of the World Online [63]. The data on the use of the two species were gathered in a Microsoft Excel sheet. For *B. erucago*, the mean age of respondents was 66 (median 65, minimum 30, maximum 85; 48 females, 25 males). For *S. inermis*, it was: 68 (median 70, minimum 34, maximum 87; 16 females, 8 males).

## Figures and Tables

**Table 1:** Use of *Bunias erucago* in Dalmatia.

Island or Region	Place	Number of Interviews	Folk Name	Preparation	Researcher (Initials of the Authors)	Date
SDC	Cista Provo (Imotska Krajina)	2	grzdulja	in boiled wild vegetable mix	TNR	2021
SDC	Dicmo (Cetinska Krajina)	1	grzdulja	cultivated, in boiled wild vegetable mix	MJD	2016
SDC	Kraj (Dicmo, Cetinska Krajina)	1	grzdulja	boiled	TNR	2021
SDC	Osoje (Dicmo, Cetinska Krajina)	1	grzdulja	in boiled wild vegetable mix	TNR	2021
SDC	Rošca (Donji Dolac, Poljica)	1	grzdulja	boiled	KD, MJD, ŁŁ	2015
SDC	Imotski (Imotska Krajina)	1	grzdulja	in boiled wild vegetable mix	TNR	2022
SDC	Vinjani Gornji (Imotski, Imotska Krajina)	2	grzdulja	in boiled wild vegetable mix	TNR	2022
SDC	Klis	1	grzdulja—velika žutinica	in boiled wild vegetable mix	TNR	2022
SDC	Ljubitovica (Munizi)	1	grzdulja	in boiled wild vegetable mix	TNR	2021
SDC	Lovreć (Imotska Krajina)	1	grzdulja	in boiled wild vegetable mix	TNR	2023
SDC	Otok (Cetinska Krajina)	1	grzdulja	boiled with potatoes	TNR	2022
SDC	Podbablje (Imotska Krajina)	5	grzdulja	boiled alone with dried meat or in a wild vegetable mix	TNR	2022
SDC	Bakovići (Primorski Dolac, Dalmatinska Zagora)	1	grzdulja	boiled	TNR	2021
SDC	Runovići (Imotska Krajina)	3	grzdulja	in boiled wild vegetable mix, cultivated	TNR	2022
SDC	Seoca (Poljica)	2	grzdulja	leaves and roots were boiled with dried meat, used mainly in autumn (Sept to Nov), better than cabbage, cultivated	KD, MJD, ŁŁ	2015
SDC	Šestanovac	2	grzduja	boiled	IVK	2018
SDC	Nečaj (Srijane, Poljica)	1	grzdulja	as a delicatessen with dried meat, seeds sown on purpose	KD, MJD, ŁŁ	2015
SDC	Vela Njiva, (Srijane, Poljica)	1	grzdulja	used in winter instead of cabbage	KD, MJD, ŁŁ	2015
SDC	Trilj (Cetinska Krajina)	1	grzdulja	in boiled wild vegetable mix	TNR	2022
SDC	Vojnić Sinjski, (Trilj, Cetinska Krajina)	1	grzdulja	in boiled wild vegetable mix	TNR	2022
SDC	Trnbusi (Poljica)	2	grzdulja	boiled	KD, MJD, ŁŁ	2015
SDC	Zagvozd (Imotska Krajina)	4	grzdulja	boiled with koštradina or other dried meat, or with mangold (blitva)	TNR	2022
SDC	Žeževica	11	grzduja	boiled	IVK	2018
SDC	Zmijavci (Imotska Krajina)	6	grzdulja	boiled alone with dried meat or in a wild vegetable mix, potatoes, a side-dish to pura (polenta) or cabbage, cultivated	TNR	2022
Krapanj	Krapanj	4	grzdulja	in boiled wild vegetable mix or with beans, especially in winter, roots also used, cultivated	IVK, ŁŁ	2018
Lopud	Lopud	1	pakoleć	in boiled wild vegetable mix	KD	2018
Mljet	Soline	1	pakoleć	in boiled wild vegetable mix	KD, ŁŁ	2017
Šipan	Šipanska Luka	3	pakoleć	in boiled wild vegetable mix	KD, ŁŁ	2018
Šipan	Suđurad	1	pakoleć	in boiled wild vegetable mix	KD, ŁŁ	2018
Korčula	Lumbarda	1	pakolić	in boiled wild vegetable mix	KD, ŁŁ	2017
DNC	Doli	1	pakoleć	in boiled wild vegetable mix	KD, ŁŁ	2013
DNC	Gromača	1	pakoleć	in boiled wild vegetable mix	KD, ŁŁ	2013
DNC	Majkovi	2	pakoleč, pakoleć	in boiled wild vegetable mix	KD, ŁŁ	2013
DNC	Mlini	1	n.d.	in boiled wild vegetable mix	KD, ŁŁ	2013
DNC	Mrčevo	2	pakoleć	in boiled wild vegetable mix	KD, ŁŁ	2013
DNC	Osojnik	2	pakoleć	in boiled wild vegetable mix	KD, ŁŁ	2013
DNC	Riđica	1	pakoleć	in boiled wild vegetable mix	KD, ŁŁ	2013
DNC	Smokovljani	1	pakoleć	in boiled wild vegetable mix	KD, ŁŁ	2013
DNC	Topolo	1	pakoleć	in boiled wild vegetable mix	KD, ŁŁ	2013
DNC	Visočani	2	pakoleć	in boiled wild vegetable mix	KD, ŁŁ	2013

Abbreviations: DNC—mainland part of Dubrovnik-Neretva County, SDC—mainland part of Split–Dalmatia County, n.d.—no data.

**Table 2:** Use of *Soda inermis* in Dalmatia.

Island	Place	No. of Interviews	Local Name	Use	Researchers (Initials of the Authors)	Year
Pag	Zubovići	1	rosica	boiled	IVK, ŁŁ	2016
Kaprije	Kaprije	3	osmukalj	only cultivated, eaten with beans	ŁŁ	2018
Krapanj	Krapanj	4	osmukalj	in boiled wild vegetable mix, also with beans, both wild and cultivated	IVK, ŁŁ	2018
Murter	Betina	1	smucanj	both wild and cultivated, boiled	IVK, ŁŁ	2018
Murter	Murter	2	smucanj	boiled alone or with beans	IVK, ŁŁ	2018
Prvić	Prvić	2	osmukalj	mainly cultivated, boiled with broad beans and fennel	IVK, ŁŁ	2018
Prvić	Prvić Luka	2	osmukalj	cultivated only, sown, sold in Šibenik market	IVK, ŁŁ	2018
Vrgada	Vrgada	2	smucanj	cooked alone, or with mangold (blitva)	IVK, ŁŁ	2018
Žirje	Žirje	2	osmukalj	both wild and cultivated, boiled in wild vegetable mix	IVK, ŁŁ	2018
Zadar County	Ražanac	1	roška	sold in the market in Zadar	ŁŁ	2018
Zlarin	Zlarin	no data	osmuhalj, osmukalj	cultivated and collected from the wild, used in a variety of dishes	[46]	2022
	Zagreb	no data	not recorded	sold in the market	[32]	2019
	Šibenik	no data	not recorded	sold in the market	[32]	2019

**Table 3:** Comparison of the use of *Soda inermis* and *Bunias erucago*.

*Bunias erucago*	*Soda inermis*	Feature
78	20	Number of use-reports from our fieldwork
Frequently	Rarely	Reported by more than one informant in the same locality
Wide (mainland and islands)	Narrow (mainly islands)	Geographic spread
Rarely cultivated	Mainly cultivated	Cultivation

## Data Availability

The raw data are included in the manuscript (Table 1 and Table 2). Further inquiries can be directed to the corresponding author.
